# A Randomized Trial Evaluating Bioimpedance Spectroscopy Versus Tape Measurement for the Prevention of Lymphedema Following Treatment for Breast Cancer: Interim Analysis

**DOI:** 10.1245/s10434-019-07344-5

**Published:** 2019-05-03

**Authors:** Sheila H. Ridner, Mary S. Dietrich, Michael S. Cowher, Bret Taback, Sarah McLaughlin, Nicolas Ajkay, John Boyages, Louise Koelmeyer, Sarah M. DeSnyder, Jamie Wagner, Vandana Abramson, Andrew Moore, Chirag Shah

**Affiliations:** 1 Vanderbilt University School of Nursing, Vanderbilt University, Nashville, TN USA; 2 Department of Biostatistics, Vanderbilt Ingram Cancer Center, Vanderbilt University Medical Center, Nashville, TN USA; 3 Department of Surgery, Alleghany General Hospital, Pittsburgh, PA USA; 4 Division of Breast Surgery, Department of Surgery, Columbia University Medical Center, New York, NY USA; 5 Section of Surgical Oncology, Mayo Clinic, Jacksonville, FL USA; 6 Department of Surgery, University of Louisville, Louisville, KY USA; 7 Faculty of Medicine and Health Sciences, Macquarie University, Sydney, NSW Australia; 8 Division of Surgery, Department of Breast Surgical Oncology, The University of Texas MD Anderson Cancer Center, Houston, TX USA; 9 University of Kansas Medical Center, Westwood, KS USA; 10 Ingram Cancer Center, Vanderbilt Medical Center, Nashville, TN USA; 11 Southeast Health Southeast Cancer Center, Cape Girardeau, MO USA; 12 Department of Radiation Oncology, Cleveland Clinic, Taussig Cancer Institute, Cleveland, OH USA

## Abstract

**Background:**

Breast cancer-related lymphedema (BCRL) represents a major source of morbidity among breast cancer survivors. Increasing data support early detection of subclinical BCRL followed by early intervention. A randomized controlled trial is being conducted comparing lymphedema progression rates using volume measurements calculated from the circumference using a tape measure (TM) or bioimpedance spectroscopy (BIS).

**Methods:**

Patients were enrolled and randomized to either TM or BIS surveillance. Patients requiring early intervention were prescribed a compression sleeve and gauntlet for 4 weeks and then re-evaluated. The primary endpoint of the trial was the rate of progression to clinical lymphedema requiring complex decongestive physiotherapy (CDP), with progression defined as a TM volume change in the at-risk arm ≥ 10% above the presurgical baseline. This prespecified interim analysis was performed when at least 500 trial participants had ≥ 12 months of follow-up.

**Results:**

A total of 508 patients were included in this analysis, with 109 (21.9%) patients triggering prethreshold interventions. Compared with TM, BIS had a lower rate of trigger (15.8% vs. 28.5%, *p* *<* 0.001) and longer times to trigger (9.5 vs. 2.8 months, *p* *=* 0.002). Twelve triggering patients progressed to CDP (10 in the TM group [14.7%] and 2 in the BIS group [4.9%]), representing a 67% relative reduction and a 9.8% absolute reduction (*p* *=* 0.130).

**Conclusions:**

Interim results demonstrated that post-treatment surveillance with BIS reduced the absolute rates of progression of BCRL requiring CDP by approximately 10%, a clinically meaningful improvement. These results support the concept of post-treatment surveillance with BIS to detect subclinical BCRL and initiate early intervention.

Breast cancer represents the most common non-cutaneous cancer among women in the US and Australia, with outcomes improving over the past several decades.^1^,^2^ With improved outcomes, increasing focus has been placed on adverse effects of treatment, including breast cancer-related lymphedema (BCRL). BCRL represents a major adverse effect of treatment, which can lead to infections, reduced arm function, and reduced quality of life.^3^ Traditionally, BCRL has been associated with more aggressive local therapies (mastectomy vs. breast-conserving surgery; axillary lymph node dissection [ALND] vs. sentinel lymph node biopsy [SLNB]; radiation therapy, including regional nodal irradiation, vs. without regional nodal irradiation), as well as systemic therapies (taxane chemotherapy).^4^,^5^ Additionally, treatment for BCRL may require therapies that are resource-intensive and costly, such as complex decongestive physiotherapy (CDP).^6^

As with many disease processes, BCRL evolves with chronic changes of BCRL, preceded by a subclinical and early stage.^7^ Previously, lymphedema was only detected clinically, but the advent of technologies such as bioimpedance spectroscopy (BIS) has allowed for subclinical detection.^7^–^9^ As such, focus has been placed on earlier detection of BCRL and subsequent intervention with non-invasive measures that are less intensive and less costly than CDP. While data to date are promising and support early detection, there remains the need for a trial comparing standard diagnostic measures with BIS that include evaluation of early intervention. Therefore, a randomized trial comparing a standard BCRL diagnostic technique (volume using circumference measurement with a tape measure [TM]) with BIS was conducted. Patients with subclinical BCRL were treated with a compression sleeve and gauntlet and then re-evaluated after 4 weeks to determine if they had progressed to requiring CDP, which in this study serves as a surrogate for clinical lymphedema. After the 4-week re-evaluation, patients were then followed-up regularly to determine progression to clinical lymphedema requiring CDP, the primary endpoint. We present the interim analysis of this study.

## Methods and Materials

Approval for the study was obtained from the Vanderbilt University Institutional Review Board (IRB) and the Vanderbilt Ingram Cancer Center Scientific Review Committee prior to participant enrollment. Study activities were conducted under the guidelines set forth in the Declaration of Helsinki. This randomized study compared post-treatment surveillance with both circumference measurements (TM volume assessment) and BIS. Presurgical inclusion criteria included women ≥ 18 years of age with histologically confirmed breast cancer (invasive or ductal carcinoma in situ [DCIS]) with planned surgery, while postsurgical inclusion criteria included stage I–III invasive breast cancer or DCIS with at least one of the following: mastectomy, axillary treatment (ALND, SLNB with greater than 6 nodes, axillary radiation), and taxane-based chemotherapy. Additional postsurgical exclusion criteria included bilateral breast surgery. Exclusion criteria included a prior history of breast cancer; neoadjuvant chemotherapy; previous radiation to the breast, chest wall, or axilla; implanted medical device; conditions known to cause swelling (excluding pregnancy, congestive heart failure, chronic/acute renal disease, cor pulmonale, nephrotic syndrome, nephrosis, liver failure or cirrhosis, pulmonary edema, and thrombophlebitis or deep vein thrombosis in the arms); previous lymphedema treatment in either arm; uncontrolled intercurrent illness; psychiatric illness that would limit compliance with the study; and known allergy to electrode adhesives or compression fabrics.

**Fig. 1:**
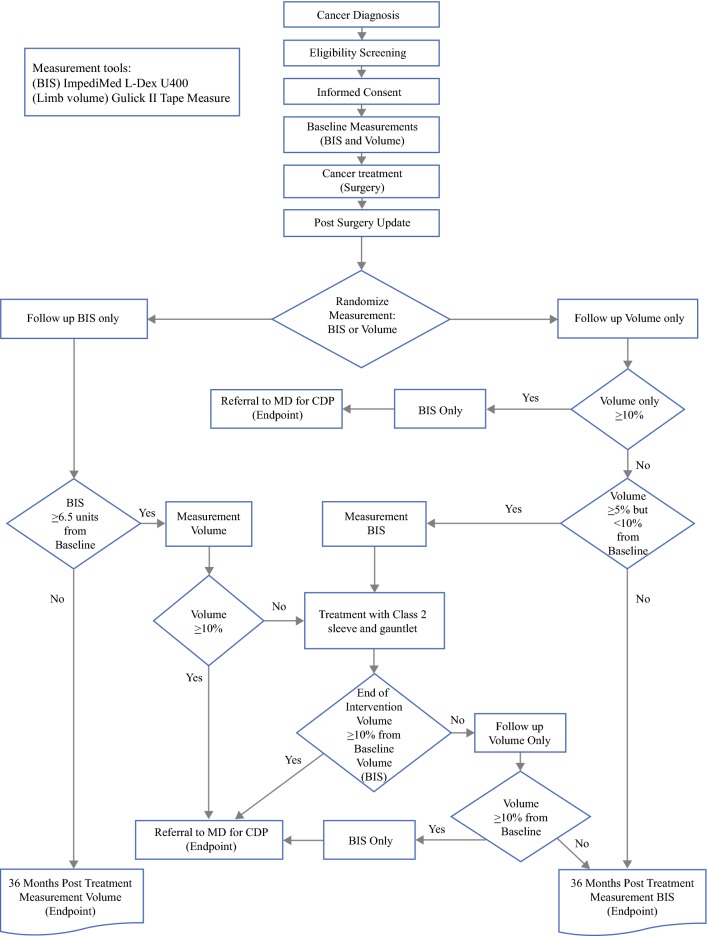
Trial schema. *BIS* bioimpedance spectroscopy, *CDP* complex decongestive physiotherapy

The trial design is presented in Fig. 1. Following consent, patients underwent a baseline presurgical measurement with BIS (L-Dex U400, Impedimed) and volume (circumference) measurements (Gulick II tape measure).^10^ Following surgery, patients were then randomized to TM versus BIS. Postsurgical assessment visits were designed to coincide with regularly scheduled clinic follow-up visits. Both the TM and BIS arms underwent planned postoperative assessments at 3, 6, 12, 18, 24, and 36 months (optional visits at 15 and 21 months), as well at the end of any intervention.

The primary aim of this study was to determine if subclinical detection of extracellular fluid accumulation via BIS and subsequent early intervention reduces the rate of progression to CDP relative to the rates seen using standard tape measurements.^11^ At study commencement, trigger points for implementation of a lymphedema prevention intervention were when patients in the BIS group had a BIS change that was ≥10 L-Dex units, representative of 3 standard deviations (SDs), higher than the presurgical baseline measure. Patients in the TM group triggered when they had a volume change in the at-risk arm that was between ≥ 5 and < 10% above presurgical baselines (without a similar change in the non-at-risk arm). Once triggered, patients underwent 4 weeks of wearing a class 2 (23–32 mmHg) compression sleeve and gauntlet therapy for 12 h per day (medi).

In 2016, growing data supported that early-stage clinical lymphedema was present when the BIS reading approximated to 7 L-Dex units, or, alternatively, when there was a change of ≥ 7 L-Dex units, representative of 2 SDs above the mean established in early studies.^12^,^13^ This required a re-examination of the trigger points as the original BIS trigger point for prevention intervention was above the diagnostic threshold. To further inform this re-examination, we undertook an analysis of 280 women with a presurgical baseline BIS measurement and at least one BIS measurement within the 12-months post-surgery.^12^ Our findings did not contradict the recent body of work supporting a change in the use of an absolute BIS value of ≥ 7 L-Dex units (representative of approximately 2 SDs from normative mean values of ‘0’) as indicative of early clinical lymphedema. In light of these data, as well as published studies, IRB and Scientific Review Committee approval was obtained to modify the prevention intervention trigger in this study from a ≥ 10 BIS L-Dex unit change to a ≥ 6.5 BIS L-Dex unit change to detect subclinical lymphedema. This places the subclinical lymphedema threshold just below the clinical threshold. The threshold for the TM group remained unchanged. Progression in both groups in this study is defined as a tape-measured volume difference change in the at-risk arm that is ≥ 10% above presurgical baselines (without a similar change in the non-at-risk arm at any follow-up subsequent to early intervention).

Enrollment in the study commenced in June 2014. The study protocol required that an interim analysis of lymphedema progression occurs when at least 500 participants were randomized and had completed the 12-month follow-up period. The milestone for conducting an interim analysis was reached in January 2018.

### Statistical Analyses

Study nominal and ordinal data values were summarized using frequency distributions; due to skewed distributions, continuous data were summarized using median and interquartile range (IQR). Chi-square tests of independence and Mann–Whitney tests were used to compare the distributions of the outcome time variables (e.g., months to trigger, months from trigger to progression), as well as the demographic, clinical, and baseline treatment characteristics of the 2 study groups. Logistic regression was used to conduct the single interim test of the hypothesis that detection via BIS will lead to a lower rate of lymphedema progression than the rate observed within the group using detection via TM circumferential volume. To maintain the final overall study hypothesis test alpha of *p* < 0.05, the Haybittle-Peto approach was used; the trial would be stopped if the symmetric stopping boundaries of *p* < 0.001 were met.

## Results

The interim analysis consisted of 508 newly diagnosed female patients with breast cancer who were followed-up for at least 12 months postsurgery (median 17.8 months, IQR 13–23). The median age was 58.8 years and 77% of patients (*n* *=* 389) were White (Table 1). Clinical characteristics and baseline assessments are summarized in Table 2. Median body mass index was 27.9 (IQR 24–33). The most frequently reported comorbid conditions were cardiovascular in nature (44%, *n* *=* 223), with 8.4% of patients (*n* *=* 36) having had a previous minor arm surgery (not meeting the exclusion criteria). A majority of patients were diagnosed with stage I breast cancer (56.7%, *n* *=* 288), with 39.0% (*n* *=* 198) of patients having stage II/III at baseline; the median baseline BIS measurement was 0.0 (IQR − 3 to + 3.0) L-Dex units. The median arm volume in the at-risk arm at baseline was 1943.2 mL (IQR 1685–2344), and 1949.6 mL (IQR 1667–2335) in the non-at-risk arm. Other than a single statistically significant difference in a history of digestive conditions, none of the key demographic, clinical, or baseline treatment characteristics differed between the groups (Tables 1, 2).

**Table 1:** Patient characteristics

Characteristic	Overall *N* *=* 508	Tape measurement *N* *=* 245	BIS *N* *=* 263	*p* value
Age, years (Median [IQR], *N*)	58.8 [50–67], 505	58.8 [50–66], 243	59.0 [50–68], 262	0.488
Years of education (Median [IQR], *N*)	16 [12–16], 506	16 [12–16], 244	16 [12–16], 262	0.899

	*N* = 508	*N* = 245	*N* = 263	
Race, *n* (%)				0.119
Do not care to respond	6 (1.2)	0 (0.0)	6 (2.3)	
Multi-racial or other^b^	37 (7.3)	15 (6.1)	22 (8.4)	
Asian	40 (7.9)	22 (9.0)	18 (6.8)	
Black or African American	36 (7.1)	17 (6.9)	19 (7.2)	
White	389 (76.6)	191 (78.0)	198 (75.3)	

	*N* = 507	*N* = 244	*N* = 263	
Ethnicity, *n* (%)				0.914
Do not care to respond	25 (4.9)	13 (5.3)	12 (4.6)	
Non-Hispanic or Latino	470 (92.7)	225 (92.2)	245 (93.2)	
Hispanic or Latino	12 (2.4)	6 (2.5)	6 (2.3)	

	*N* = 505	*N* = 244	*N* = 261	
Marital status, *n* (%)				0.357
Single	62 (12.3)	32 (13.1)	30 (11.5)	
Single, living with partner	16 (3.2)	7 (2.9)	9 (3.4)	
Married	367 (72.7)	182 (74.6)	185 (70.9)	
Widowed	33 (6.5)	12 (4.9)	21 (8.0)	
Separated	17 (3.4)	5 (2.0)	12 (4.6)	
Other	10 (2.0)	6 (2.5)	4 (1.5)	

	*N* = 505	*N* = 243	*N* = 262	
Employment, *n* (%)				0.138
Employed full-time	209 (41.4)	103 (42.4)	106 (40.5)	
Employed part-time	61 (12.1)	23 (9.5)	38 (14.5)	
Homemaker	43 (8.5)	25 (10.3)	18 (6.9)	
Retired	154 (30.5)	71 (29.2)	83 (31.7)	
Unemployed	7 (1.4)	6 (2.5)	1 (0.4)	
On disability benefit	6 (1.2)	4 (1.6)	2 (0.8)	
Other	25 (5.0)	11 (4.5)	14 (5.3)	

	*N* = 505	*N* = 244	*N* = 261	
Residence, *n* (%)				0.529
City/urban	120 (23.8)	53 (21.7)	67 (25.7)	
Country/rural/small town	119 (23.6)	61 (25.0)	58 (22.2)	
Suburb	266 (52.7)	130 (53.3)	136 (52.1)	

	*N* = 507	*N* = 245	*N* = 262	
Any government insurance, *n* (%)	320 (63.1)	153 (62.4)	167 (63.7)	0.763
Any non-government insurance, *n* (%)	391 (77.1)	192 (78.4)	199 (76.0)	0.518
No insurance, *n* (%)	2 (0.4)	1 (0.4)	1 (0.4)	0.962
Ever smoked, *n* (%)	162 (32.0)	84 (34.3)	78 (29.8)	0.276
Ever drank alcohol, *n* (%)	361 (71.2)	174 (71.0)	187 (71.4)	0.930

*BIS* bioimpedance spectroscopy, *IQR* interquartile range

^a^All participants indicated female sex

^b^Including American Indian, Alaskan Native, Native Hawaiian, Pacific Islander, Aboriginal, Torres Strait Islander

**Table 2:** Baseline clinical characteristics and assessments

Characteristic	Overall *N* *=* 508	Tape measurement *N* *=* 245	BIS *N* *=* 263	*p* value
Medications^a^				
*β*-Blockers	40 (8.0) [500]	19 (7.9) [241]	21 (8.1) [259]	0.926
Diuretics	59 (11.7) [506]	23 (9.4) [245]	36 (13.8) [261]	0.123
Oral steroids	12 (2.4) [505]	5 (2.0) [244]	7 (2.7) [261]	0.641
NSAIDs	116 (22.9) [506]	48 (19.6) [245]	68 (26.1) [261]	0.084
Most commonly reported history of comorbid conditions^a,b^				
Cardiovascular	223 (44.0) [507]	106 (43.3) [245]	117 (44.7) [262]	0.752
Skeletal	165 (32.5) [507]	80 (32.7) [245]	85 (32.4) [262]	0.960
Endocrine	123 (24.3) [507]	53 (21.6) [245]	70 (26.7) [262]	0.182
Digestive	119 (23.5) [507]	71 (29.0) [245]	48 (18.3) [262]	**0.005**
Integumentary	107 (21.1) [507]	52 (21.2) [245]	55 (21.0) [262]	0.949
History of surgery^a,b^				
Any	437 (86.2) [507]	208 (84.9) [245]	229 (87.4) [262]	0.414
Arm	36 (8.4) [431]	14 (6.9) [204]	22 (9.7) [227]	0.289
Shoulder	17 (3.9) [431]	7 (3.4) [204]	10 (4.4) [227]	0.604
Truncal	85 (19.7) [432]	44 (21.5) [205]	41 (18.1) [227]	0.374
Stage of cancer^a^				0.227
0 (DCIS)	22 (4.3)	12 (4.9)	10 (3.8)	
I	288 (56.7)	129 (52.7)	159 (60.5)	
II	164 (32.3)	89 (36.3)	75 (28.5)	
III	34 (6.7)	15 (6.1)	19 (7.2)	
Baseline assessments^c^				
BIS	0.0 [−3 to 3] (508)	−0.1 [−3 to 3] (245)	0.0 [−3 to 3] (263)	0.959
Arm volume				
At-risk arm, mL	1943.2 [1685–2344] (508)	1929.4 [1686–2360] (245)	1971.6 [1682–2323] (263)	0.533
Non-at-risk arm, mL	1949.6 [1667–2335] (508)	1947.5 [1668–2334] (245)	1958.2 [1667–2337] (263)	0.698
Percentage difference	0.2 [−2 to 4] (508)	0.4 [−2 to 3] (245)	0.2 [−3 to 4] (263)	0.629
BMI	27.9 [24−33] (507)	28.3 [24–33] (244)	27.9 [24–33] (263)	0.809
Number of skin conditions				
At-risk arm	0.0 [0–1] (508)	0.0 [0–1] (245)	0.0 [0–1] (263)	0.911
Non-at-risk arm	0.0 [0–1] (507)	0.0 [0–1] (244)	0.0 [0–1] (263)	0.901

Bold value indicates statistical significance (*p* < 0.05)

*NSAIDs* non-steroidal anti-inflammatory drugs, *BIS* bioimpedance spectroscopy, *DCIS* ductal carcinoma in situ, *BMI* body mass index, *IQR* interquartile range

^a^Data are expressed as *N* yes (% yes) [*N* responses]

^b^Besides current breast cancer

^c^Data are expressed as median [IQR] (*N*)

Table 3 presents the treatment characteristics for the cohort. With respect to breast surgery, 78.9% (*n* *=* 401) of patients underwent breast-conserving surgery only; of those with total mastectomies, 83.2% (*n* *=* 89/107) had reconstruction. Furthermore, 97.0% (*n* *=* 492) had some form of axillary surgery (ALND, SLNB, or both). Overall, 42.0% (*n* *=* 213) of patients received chemotherapy, with the majority receiving only adjuvant chemotherapy (*n* *=* 164, 77.0%), and 78.3% (*n* *=* 397) of patients received endocrine therapy. Overall, 430 patients (84.6%) received radiation therapy, with 75.4% (*n* *=* 316) receiving a tumor bed boost and 22.3% (*n* *=* 95) receiving regional nodal irradiation. Looking at the overall treatment provided, 8.5% (*n* *=* 43) of patients received surgery only, 50.5% (*n* *=* 254) received surgery and radiation therapy, 7.1% (*n* *=* 36) received surgery and chemotherapy, and 33.9% (*n* *=* 171) received surgery, chemotherapy, and radiation therapy. The groups were very similar in terms of the specific criteria used for study inclusion. The most commonly occurring criterion was radiation to the chest wall or breast (83.8%, *n* *=* 425; BIS 85.6%, TM 82.0%), followed by a taxane type of chemotherapy (38%, *n* *=* 192; BIS 36.5%, TM 39.3%). Approximately 50% of patients in both groups met multiple inclusion criteria (48.8%, *n* *=* 248; BIS 47.5%, TM 50.2%).

**Table 3:** Breast treatment* characteristics

Treatment characteristics	Overall *N* *=* 508	Tape measurement *N* *=* 245	BIS *N* *=* 263	*p* (value)
Type of surgery, *n* (%)				
Breast conservation	401 (78.9)	189 (77.1)	212 (80.6)	0.338
Mastectomy	107 (21.1)	56 (22.9)	51 (19.4)	
Reconstruction, *n* (%)^a^	89 (17.6)	46 (18.8)	43 (16.4)	0.485
Node dissection, *n* (%)	493 (97.0)	239 (97.6)	254 (96.6)	0.517
Dissection type^b^				0.392
ALND only	46 (9.3)	22 (9.2)	24 (9.4)	
SLNB only	377 (76.6)	176 (73.9)	201 (79.1)	
ALND and SLNB	59 (12.0)	34 (14.3)	25 (9.8)	
Other^c^	10 (2.0)	6 (2.5)	4 (1.6)	
SLNB only				0.291
<6 nodes	362 (96.0)	167 (94.9)	195 (97.0)	
≥6 nodes	15 (4.0)	9 (5.1)	6 (3.0)	
Total number of nodes dissected (Median [IQR], *N*)	3.0 [2–5], 493	3.0 [2–5], 239	3.0 [2–4], 254	0.090
Total number of positive nodes (Median [IQR], *N*)	0.0 [0–1], 493	0.0 [0–1], 239	0 [0–0], 254	0.269

	*N* = 507	*N* = 244	*N* = 263	*p* value
Chemotherapy, *n* (%)	213 (42.0)	106 (43.4)	107 (40.7)	0.530
Neoadjuvant	24 (11.3)	11 (10.4)	13 (12.1)	0.893
Adjuvant	164 (77.0)	83 (78.3)	81 (75.8)	
Both	25 (11.7)	12 (11.3)	13 (12.1)	
Chemotherapy type (if received)				0.836
Any taxane	192 (90.1)	96 (90.6)	96 (89.7)	
Other (non-taxane)	21 (9.9)	10 (9.4)	11 (10.3)	
Radiation therapy, *n* (%)	430 (84.6)	203 (82.9)	227 (86.3)	0.280
Boost^d^	316 (75.4)	151 (76.3)	165 (74.7)	0.704
Radiation location				
Breast/chest wall	425 (99.8)	200 (99.5)	225 (100.0)	0.289
Regional nodes	95 (22.3)	45 (22.4)	50 (22.2)	0.967

	*N* = 507	*N* = 244	*N* = 263	*p* value
Endocrine therapy, *n* (%)	397 (78.3)	181 (74.2)	216 (82.1)	**0.030**

	*N* = 503	*N* = 245	*N* = 258	*p* value
Complete treatment, *n* (%)				0.798
Surgery	43 (8.5)	24 (9.8)	19 (7.4)	
Surgery + radiotherapy	254 (50.5)	117 (47.8)	137 (53.1)	
Surgery + chemotherapy (taxane)	34 (6.8)	19 (7.8)	15 (5.8)	
Surgery + chemotherapy (not taxane)	2 (0.4)	1 (0.4)	1 (0.4)	
Surgery + radiotherapy + chemotherapy (taxane)	153 (30.4)	76 (31.0)	77 (29.8)	
Surgery + radiotherapy + chemotherapy (not taxane)	17 (3.4)	8 (3.3)	9 (3.5)	

Bold value indicates statistical significance (*p* < 0.05)

All participants underwent surgery

*BIS* bioimpedance spectroscopy, *ALND* axillary lymph node dissection, *SLNB* sentinel lymph node biopsy, *IQR* interquartile range

^a^*N* *=* 507; BIS *N* *=* 262

^b^One type of axillary surgery was unknown, *N* *=* 492

^c^Including interpectoral, intramammary, non-sentinel, and unknown type

^d^*N* *=* 419; tape measurement *N* *=* 198, BIS *N* *=* 221

Data for triggered events and progression to CDP for the interim sample are presented in Table 4. Of the 508 patients, 10 (6 in the TM group and 4 in the BIS group) progressed either at their initial post-randomization visit or between other study visits before an intervention could be instituted. Of the remaining 498 patients, statistically significantly fewer BIS participants triggered an intervention (15.8% BIS vs. 28.5% TM; *p* = 0.001). When including the 10 patients who progressed and were excluded from the primary analysis, the trigger rates were 15.6% BIS vs. 27.8% TM (*p* = 0.001). Within the BIS group, 26.8% triggered with the updated L-Dex criteria (*n* *=* 11/41), while the rest triggered when using the initial ≥ 10 criteria. Furthermore, the time from randomization to trigger in the BIS group was significantly longer than the TM group (median 2.8 months TM vs. 9.5 months BIS, *p* = 0.002) (Table 4). Overall, not including those who progressed without intervention and thus did not meet the inclusion criteria for the endpoint analysis, progression was observed in only 11.0% (*n* *=* 12) of those patients who triggered an intervention, therefore sample sizes were quite small. In the BIS group, 4.9% (2/41) progressed, while in the TM group, 14.7% (10/68) progressed. The difference between the rates of progression to CDP were not statistically significant (*p* = 0.130); this does not meet the stopping criteria set forth in the protocol. The median time to progression was 6.0 months (IQR 0.8–16.9), with similar times for the circumference and BIS groups (median 6.0 and 6.7 months, respectively; *p* *=* 0.389) (Table 4).

**Table 4:** Summary of trigger and progression, by surveillance group

		Tape measurement	BIS
	*N* *=* 508	*N* *=* 245	*N* *=* 263
Follow-up (months)^a^	17.8 (13–23)	17.6 (13–23)	18.2 (13–23)
Progressed before intervention^b^	10 (2.0)	6 (2.4)	4 (1.5)

	*N* *=* 498	*N* *=* 239	*N* *=* 259	*p*-Value
Sample for potential trigger				
Triggered^b^	109 (21.9)	68 (28.5)	41 (15.8)	**0.001**
Median months to trigger^a^	4.0 (1.0–11.2)	2.8 (0.6–5.6)	9.5 (2.3–10.1)	**0.002**
Progression^b^	12 (11.0)	10 (14.7)	2 (4.9)	0.130
Median time, months [trigger to progression]^c^	6.0 (0.8, 16.9)	6.0 (1.4, 16.9)	6.7 (0.8, 12.5)	0.389

Bold values indicate statistical significance (*p* < 0.05)

Months from randomization to last assessment for these analyses

Progression to criteria for being off-study without the interim intervention (subclinical) threshold

*BIS* bioimpedance spectroscopy, *IQR* interquartile range

^a^Median (IQR)

^b^*n* (%)

^c^Median (minimum, maximum)

## Discussion

The interim results from this large, randomized trial demonstrate several key findings. First, outcomes addressing the primary endpoint demonstrate an approximate 10% reduction in the rates of CDP with the use of BIS surveillance compared with TM (4.9% vs. 14.7%), representing a 67% relative reduction. While not statistically significant, it is important to recognize that this is an interim analysis with only 500 completing 12 months of follow-up. As such, the number of events are low, but it is expected that with greater numbers of enrolled patients and longer follow-up, additional events will occur and serve to support and strengthen these initial findings. If current rates remain consistent, it is expected that with the greater number of events, the difference between BIS and TM will become statistically significant. These findings are similar to other studies where initial outcomes were not significant due to the low numbers of events, but, with greater follow-up and events, differences became statistically significant.^14^ In the ATAC trial (comparing tamoxifen vs. anastrazole vs. combined treatment), a clinically significant benefit was seen, even though no statistical difference in breast cancer mortality (12% absolute reduction) was noted due to the lower number of events.^15^ The results of the current analysis can be interpreted in the same fashion as they are likely clinically significant, while not reaching statistical significance at this time due to the small number of events (progression to CDP).^14^

The second major finding of this trial was the rate of ‘triggers’ in each group. Initially, due to the change in the subclinical threshold criteria, concerns existed regarding potential false positive readings with BIS.^16^ However, the interim results reflect a lower rate of triggers in the BIS arm compared with TM (28.5% TM vs. 15.8% BIS). Additionally, the median time to trigger was considerably earlier in the TM group compared with the BIS group (TM median approximately 3 months postsurgery; BIS median approximately 10 months postsurgery). Although the reason for this difference in the time to trigger is currently unknown, we do know that TM detects a change in the whole arm volume, while BIS only detects an extracellular fluid change. It is possible that at 3 months postsurgery there remains, in some patients, a generalized, whole arm inflammatory response that is identified by TM. Increased extracellular fluid may not be a major factor in that volume change. Moving forward, future analyses for this study should evaluate factors associated with triggering for both groups. If the lower rate of triggers, as well as the lower rates of CDP, persist for the BIS arm, this suggests BIS may also be more specific than TM measurements, reducing the rate of false positives. This would mean that BIS could even be more cost effective than TM as there would be fewer patients requiring CDP (based on interim analysis) and fewer lymphedema diagnostic evaluations would be indicated.

### Supporting Data

It should be noted that a 10% absolute reduction in CDP represents a clinically meaningful outcome as CDP represents a time-consuming, resource-intensive, and costly treatment.^6^ For the trial, CDP was considered a surrogate for chronic BCRL. In comparison, the EORTC 10981-22023 AMAROS (radiotherapy or surgery of the axilla after positive sentinel node in breast cancer) randomized trial (which included a similar patient cohort as the current trial) identified a 23% rate of chronic BCRL at 5 years, with rates of 15–25% noted in other series.^17^–^19^ As such, the results of the present trial (although with a shorter follow-up) are promising, with only a 4.9% rate of CDP use in the BIS group.

To date, studies evaluating diagnostic and therapeutic modalities for BCRL have been limited by small patient numbers and limited follow-up. Many were performed in an era when all patients underwent an axillary dissection with associated higher rates of lymphedema. Two previously reported randomized trials evaluating early detection have been performed, but were limited by these issues as well as the lack of using a high-sensitivity diagnostic modality.^20^,^21^ The current trial represents one of the largest BCRL trials to date and is one of the few randomized trials evaluating diagnostic and therapeutic interventions. The postsurgical inclusion criteria are believed to capture patients at risk for lymphedema, while allowing findings to be generalized to the breast cancer survivor population as a whole. Taken in this context, these preliminary results are important and support the use of subclinical detection with BIS and early intervention for patients with breast cancer at risk for lymphedema.

### Statistical Considerations

At this time, the stopping criteria (*p* *=* 0.001) were not met. This is not surprising given that only 109 (approximately 22%) of the total sample of 498 patients triggered early intervention, and only 12 (11%) of those 109 patients have progressed. At this time, proposed enrollment has been completed and follow-up of all patients will continue for the complete study period.

Furthermore, 10 participants progressed immediately postsurgery or between assessment visits, requiring withdrawal from the study, and were therefore not included in these analyses. These cases suggest that, although vital, standard lymphedema assessment at routine clinical follow-ups may miss early swelling in some patients. This raises questions about the potential value of home self-monitoring post surgery as a prevention strategy, especially in patients at high risk for developing lymphedema.

## Conclusions

The results of this interim analysis demonstrate that patients undergoing surveillance with BIS had reduced but non-statistically significant reductions in the rates of progression requiring CDP compared with TM. These results are currently supportive of the need for subclinical detection and early intervention for patients with BCRL, with a 10% absolute reduction and 67% relative reduction in the rates of CDP. Further data with a longer follow-up than in this study is expected in the years to come and will strengthen these early, positive, practice-changing results.
